# Bioinspired MXene-Based User-Interactive Electronic Skin for Digital and Visual Dual-Channel Sensing

**DOI:** 10.1007/s40820-022-00838-0

**Published:** 2022-05-03

**Authors:** Wentao Cao, Zheng Wang, Xiaohao Liu, Zhi Zhou, Yue Zhang, Shisheng He, Daxiang Cui, Feng Chen

**Affiliations:** 1 Department of Orthopedic, School of Medicine, Spinal Pain Research Institute, Shanghai Tenth People’s Hospital, Tongji University, Shanghai, 200072 People’s Republic of China; 2 National Engineering Research Center for Nanotechnology, Shanghai, 200241 People’s Republic of China; 3 Institute of Micro-Nano Science and Technology, School of Electronic Information and Electrical Engineering, Shanghai Jiao Tong University, Shanghai, 200240 People’s Republic of China

**Keywords:** MXene, Electronic skin, Electromechanical behavior, Joule heating, Visualization

## Abstract

User-interactive electronic skin (e-skin) that could convert mechanical stimuli into distinguishable outputs displays tremendous potential for wearable devices and health care applications. However, the existing devices have the disadvantages such as complex integration procedure and lack of the intuitive signal display function. Here, we present a bioinspired user-interactive e-skin, which is simple in structure and can synchronously achieve digital electrical response and optical visualization upon external mechanical stimulus. The e-skin comprises a conductive layer with a carbon nanotubes/cellulose nanofibers/MXene nanohybrid network featuring remarkable electromechanical behaviors, and a stretchable elastomer layer, which is composed of silicone rubber and thermochromic pigments. Furthermore, the conductive nanohybrid network with outstanding Joule heating performance can generate controllable thermal energy under voltage input and then achieve the dynamic coloration of silicone-based elastomer. Especially, such an innovative fusion strategy of digital data and visual images enables the e-skin to monitor human activities with evermore intuition and accuracy. The simple design philosophy and reliable operation of the demonstrated e-skin are expected to provide an ideal platform for next-generation flexible electronics.

**Supplementary Information:**

The online version contains supplementary material available at 10.1007/s40820-022-00838-0.

## Highlights

- A bioinspired MXene-based user-interactive electronic skin (e-skin) for digital and visual dual-signal sensing was designed and fabricated.
- The MXene-based e-skin exhibited an excellent electromechanical sensing performance and realized the real-time monitoring of human activities, such as handwriting, drinking, walking, and speaking.
- Benefiting from the outstanding Joule-heating performance of MXene-based film, the e-skin with thermochromic pigments could realize a wider range and dynamic coloration for passive displays and visual recognition of various human motions.

**Supplementary Information:**

The online version contains supplementary material available at 10.1007/s40820-022-00838-0.

## Graphical abstract

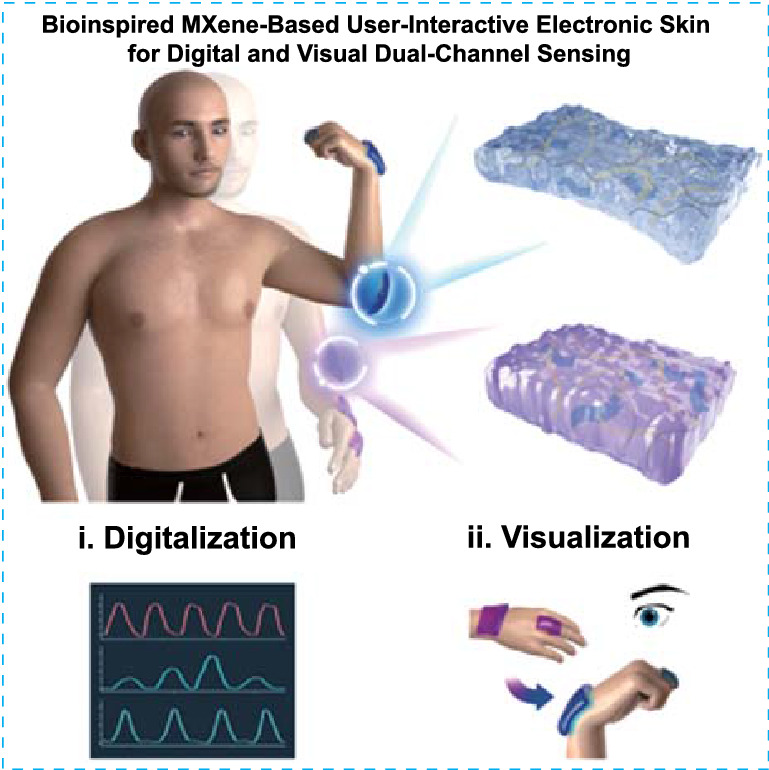

## Introduction

Human skin, as a natural and remarkable integrated sensor network, can transduce environmental stimuli (i.e., tension, pressure, temperature, and vibration) into electrical signals, which are then processed by the brain for the generation of effective instruction. Inspired by this multisensory feature of biological skin [1–3], electronic skins (e-skins) with similar sensibilities are proposed and gradually developed into an effective interactive medium for numerous novel applications, such as artificial prosthetics [4–6], health monitoring [7–10], wearable devices [11, 12], and next-generation user interfaces for augmented reality [13–15]. To obtain high-performance e-skin that imitates or even outperforms biological skin, new materials and rational manufacturing methods are developed for the construction of integrated electronic devices [16, 17]. Recent advances in two-dimensional (2D) early-transition metal carbides/carbonitrides (MXenes) suggest that MXenes have been considered as a thriving conductive agent in next-generation e-skin sensors [18–21], attributed to their great hydrophilicity and high electronic conductivity [22–26]. For example, Dong and co-workers reported a multifunctional e-skin system with broad working range that integrates MXene with vinyl silica nanoparticle–polyacrylamide hydrogel through the bridging action of polypyrrole nanowires layer [27]. Shen and colleagues utilized a vacuum filtration technology by coupling few-layer MXene with a polyacrylonitrile network for the fabrication of a stable e-skin with excellent pressure sensing performance that could realize the rapid monitoring of human physiological activities [18]. However, despite these achievements, the above works on MXene-based e-skins only focus on the optimization of devices for sensing abilities but lack research on another crucial function, namely visual recognition.

Contrasting to human skin, some animals’ skin displays additional function [28, 29], for example, the cephalopod’s skin possesses color-changing abilities [30–32]. The cephalopods change their skin color by regulating the arrangement of reflective plates inside iridophores [33]. Recently, many innovative works that mimicking the color-switching abilities of animals for the achievement of multifunctional visualization devices, which can translate mechanical stimuli into intuitive visual signals, have been widely reported [34–36]. For example, Chen et al. integrated a bimodal artificial sensory neuron for the implementation of the visual-haptic fusion [37]. The optic and pressure information could be first collected and converted into electrical signals by photodetector and pressure sensor and then transmitted to the synaptic transistor through an ionic cable to realize the multimodal sensory fusion. Wang et al. developed a nanowire light-emitting diode (LED)-based pressure sensor by utilizing the piezo-phototronic effect of ZnO/GaN nanowire LEDs for the conversion of the mechanical stress input into the optical output [38]. In these devices, the mechanical sensing information and visual signals could be obtained simultaneously by using multi-component integrated electronics, which require an extremely complicated fabrication procedure. Even though some promising advances have been gained, the exploration of simple device structures to achieve a high-efficiency multimodal fusion is still urgently required.

Here, we develop an ultra-flexible and user-interactive e-skin that fuses electromechanical/digital data and visual images for human activities recognition tasks. This bioinspired e-skin consists of two core components: a conductive strain-sensing layer and a stretchable silicone-based thermochromic layer. For the strain-sensing layer, we construct a functional nanomaterials-integrated network, which comprises carbon nanotubes (CNTs), cellulose nanofibers (CNFs), and MXene nanosheets. Benefiting from the addition of conductive CNTs/CNFs/MXene (CCM) film, the e-skin can convert the external strain stimuli into electrical signals in a way that resemble the role of a sensory nerve in human skin. In addition, the CCM film also possesses an excellent Joule heating performance and can deliver thermal energy to the thermochromic pigments within silicone rubber to realize dynamic coloration for passive displays and military camouflage. Notably, this concept is simple, general, and scalable, and avoids the complex construction procedures of integrated circuits and functional electron devices. Especially, CCM e-skin can not only recognize the mechanical strain with quantification through electrical signals but also display the degree of strain with more intuition via optical signals. These features demonstrate that the CCM e-skin provides a new platform for visual monitoring of human motions with potential application in autonomous artificial intelligence, skin prosthesis, and health care devices.

## Experimental Section

### Materials

Ti_3_AlC_2_ (MAX phase) powders were purchased from Jilin 11 Technology Co., Ltd. Lithium fluoride (LiF, ≥ 99%) and sodium hypochlorite (NaClO) were obtained from Aladdin Industrial Corporation. Sodium hydroxide (NaOH) and hydrochloric acid (HCl, 36 ~ 38 wt%) were purchased from Sinopharm Chemical Reagent Co., Ltd. The CNTs (diameter: ~ 30 nm) were provided by Shenzhen Nanotech Co., Ltd., of China. Thermochromic dyes were obtained from Shenzhen Qiansebian Pigments Co., Ltd. All the starting materials were utilized without further purification.

### Synthesis of Ti_3_C_2_ MXene Nanosheets

Ti_3_C_2_ MXene nanosheets used in this article were produced according to a modified HCl/LiF method [39]. Briefly, 1 g of Ti_3_AlC_2_ powders (particle size ≤ 38 μm) was gradually added to 20 mL of the etchant solution which contained 1.6 g of LiF and 20 mL of 9 M HCl. The mixture was stirred continuously at 50 °C for 30 h. After a complete reaction, the resulting product was washed with deionized (DI) water for five times, which involved 5 min of centrifugation at 3500 rpm for each time. As the pH of the mixture reached almost neutral, the collected sediment was redispersed to DI water and sonicated under an ice bath for 20 min to delaminate the clay‑like MXene. The self-delaminated MXene was then centrifuged at 1500 rpm for 30 min to remove the unexfoliated MAX sediment, and the supernatant was collected. The resulting dispersion was subsequently centrifuged for 20 min at 4500 rpm, after which a dark-green supernatant solution of Ti_3_C_2_ MXene was observed and then collected. The obtained MXene nanosheets dispersion was sealed and stored at ~ 4 °C.

### Preparation of TEMPO‑Mediated Oxidized CNFs

CNFs were prepared following a well-established protocol as previously described [40, 41]. 1 g of softwood pulp was added into a reaction solution (100 mL) containing TEMPO (0.1 × 10^−3^ M) and NaBr (1 × 10^−3^ M) under continuous stirring. Subsequently, NaClO solution (5 mmol g^−1^) was dropwise added to the above mixture to initiate oxidation reaction. Whereafter, the pH value of the mixture was maintained at approximately 10 during the preparation by adding 1 M NaOH solution. To obtain the purified fibers, TEMPO‑oxidized pulp were washed repeatedly with DI water. And the nanofibers could be obtained after a vigorous stirring of the purified pulp fibers for about 20 min. The unfibrillated precipitates were removed from the nanofibers dispersion after a high-speed centrifugation with 10,000 rpm for 30 min. Last, the collected supernatant was further passed through a high-pressure homogenizer to acquire the homogeneous CNFs dispersion.

### Fabrication of CCM Film

CNTs powder was added into the CNFs dispersion with a CNTs/CNFs weight ratio of 10:1. And the dispersion process was conducted by vigorous shaking and strong sonication for 20 min. Afterward, MXene nanosheets dispersion with various solid content (0.2, 0.5, and 1 mg) and CNTs/CNFs dispersion were mixed under stirring to generate a homogeneous CCM mixture. To yield CCM films, the as-obtained CCM mixture was vacuum filtered through a filter membrane and subsequently sandwiched between two hot platens at 60 °C for 20 min.

### Construction of User-Interactive CCM E-Skin

The CCM e-skin was fabricated via a typical mold casting process. Typically, components A and B of the liquid silicone rubber (Ecoflex 00–30) were blended at a weight ratio of 1:1, gently stirred for about 10 min. Subsequently, the thermochromic dyes were slowly added into the liquid silicone rubber under continuous stirring and then placed in a vacuum drying chamber for approximately 10 min to eliminate bubbles. The liquid silicone rubber with thermochromic dyes was then prepolymerized in a PTFE mold at room temperature for 1 h. The as-prepared CCM film was cut into rectangles (1.0 × 2.0 cm^2^) and then transferred to the prepolymerized silicone rubber substrate with the CCM film downward. The cellulose filter membrane could be broken down in acetone within 30 min. Afterward, the conductive copper wires were coated on both ends of the CCM film surface. Finally, liquid silicone rubber mixed with various thermochromic pigments was employed for the encapsulation and construction of user-interactive CCM e-skin.

### Characterization

SEM (Hitachi S-4800) and EDS were conducted to investigate the surface morphologies and elemental dispersion of samples. The as-prepared MXene nanosheets, CNFs, and CNTs were characterized using HR-TEM (JEM-2100F). The XRD patterns of the samples were measured by an X-ray diffractometer with Cu Kα radiation (λ = 1.54178 Å). The chemical components of MXene were analyzed by using an ESCALAB 250Xi (Thermo Scientific, UK). The functional group of CNFs were investigated via an FTIR spectrometer (FTIR-7600, Lambda Scientific, Australia). AFM (Asylum Research) was utilized to characterize the thickness of samples. The size distribution of the samples was obtained using a Nano ZS90 laser particle analyzer (Malvern Instruments, UK). The viscoelastic properties of the samples were studied using a rheometer (Physica MCR301). A physical property measurement system (Quantum Design) was employed to measure the electrical conductivity of the samples.

### Molecular Dynamics Simulation

A carbon nanotube model (length: 7.8704 nm; diameter: 2.711 nm) was built using Visual Molecular Dynamics (VMD) software. Then, the obtained nanotube was placed in a rectangle box with dimensions of 8 × 8 × 7.8704 nm^3^. The nanotube was placed in the center of the box with its central axis extending along the z-axis of the box. Subsequently, 20 glucose molecules were randomly placed around the nanotube. Finally, except for the inner region of the nanotube, the box was filled with water molecules. The simulation process was conducted using the GROMACS package (version 2019.3) with the CHARMM all-atom force field. The atomic charges of the atoms were generated using the CGenFF program (version 1.0.0 and force field version 3.0.1). Firstly, the steep descent method was used to minimize the energy of the system. Water molecules were described by the TIP3P models. Subsequently, molecular dynamics simulations under the NVT ensemble at 298 K were performed for 100 ns. LINCS algorithm was used to constrain the bond lengths of other components. The temperature was maintained using the V-rescale thermostat algorithm. The cutoff distance for the Lennard–Jones and electrostatic interactions was 1.2 nm. Last, the long-range electrostatic interactions could be obtained by employing the particle mesh Ewald method. A Molecular Dynamics software was used to make the configurations visually.

### Strain Sensing Tests

A universal testing machine (HY-940FS) combined with a software processing system (TM2101) was employed to conduct the strain sensing measurements. The resistance variation of samples could be calculated by employing an electrochemical workstation (CS350H) by maintaining a constant input voltage of 1 V on the two sides of the CCM e-skin to record the real-time current signal. To assess the resistance changes of CCM materials during various joint movements, the CCM e-skin was attached directly to the surface of the skin. All participant experiments with human subjects were standardized with the informed consent of the volunteers and approved by the Ethical Committee of Shanghai Tenth People’s Hospital of Tongji University School of Medicine.

### Joule-Heating Performance and Visual Analysis

The electrothermal behavior of the as-prepared CCM e-skins was investigated using a DC power supply (MS-3010D). An IR thermal imaging camera (FLIR A325SC) was performed to record the thermal images and temperature data of the samples. The color display of the CCM e-skins could be driven by tuning the output power of the power supply machine.

## Results and Discussion

### Fabrication of Digital-Visual Fusion CCM E-Skin

**Fig. 1:**
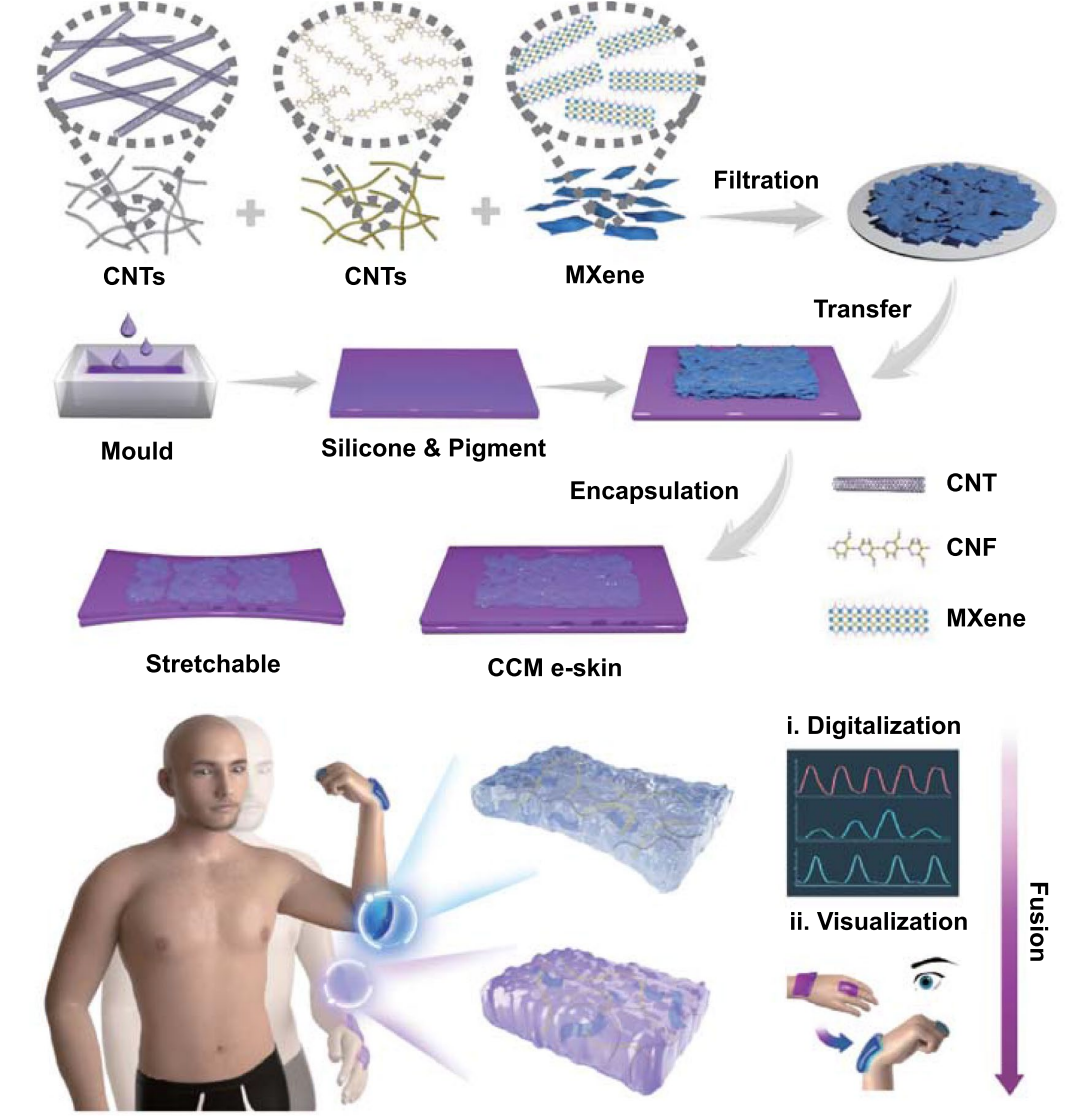
Schematic illustration of the preparation of digital-visual fusion CCM e-skin. CNTs, CNFs, and Ti_3_C_2_ MXene nanosheets were firstly mixed and filtrated to obtain the CCM film. Subsequently, a sticky substrate was fabricated after the precuring of silicone and pigment with the assistance of a PTFE mold. Finally, the CCM film was transferred onto the silicone and pigment substrate and further encapsulated to construct a flexible and user-interactive CCM e-skin, where the CCM layer served as the strain sensing and Joule heating layer, and the silicone and pigment substrate served as the thermochromic component and encapsulation layer. Thus, the CCM e-skin could utilize the digital-visual fusion to realize the intelligent monitoring of human motions

We produced a flexible, strain sensitive, and user-interactive CCM e-skin by transferring the conductive CCM layer inside the silicone rubber (Fig. 1). The fabrication process encompassed three steps: First, the CCM film was formed after a vacuum-assisted filtration process of CNTs/CNFs/MXene nanocomposites; second, liquid silicone rubber doped with thermochromic pigments was prepolymerized to form a sticky silicone and pigment film by using the polytetrafluoroethylene (PTFE) mold; last, the CCM film was transferred onto the surface of precured silicone and pigment substrate and further encapsulated to obtain the CCM e-skin, in which the conductive CCM film served as the strain sensing and Joule heating layer, and the silicone and pigment substrate served as the thermochromic component and encapsulation layer to provide the flexibility and stretchability of CCM e-skin. The CCM e-skin could be attached to the human skin for the monitor of human activities by two artificial sensory channels: the digital and the visual channel. The electromechanical/digital channel comprised a strain sensor that could detect the human body movements by analyzing the variation of resistance signal, whereas the visual channel was mainly based on the mechanism of Joule heating and thermomechanochromism. This multimodal fusion strategy enables the CCM e-skin to study human activities with evermore intuition and accuracy.

### Characterization of CCM E-Skin

**Fig. 2:**
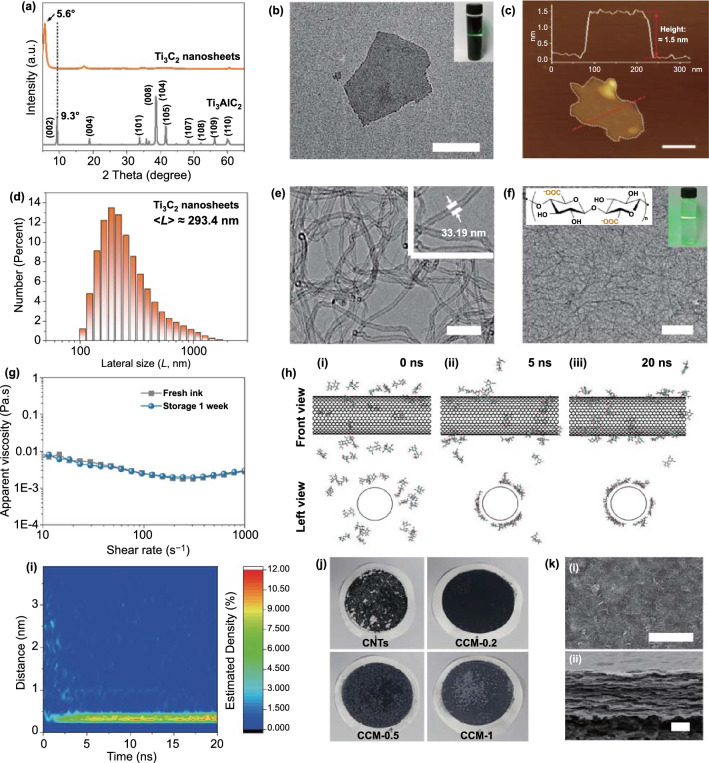
Characterization of Ti_3_C_2_ nanosheets, CNTs, CNFs, and CCM films. **a** XRD patterns of the Ti_3_AlC_2_ precursor and the as-prepared Ti_3_C_2_ nanosheets. **b** TEM image of the as-prepared 2D Ti_3_C_2_ nanosheets (scale bar: 200 nm). Inset: the photograph of Ti_3_C_2_ MXene dispersion. **c** AFM image and the height profile of the Ti_3_C_2_ nanosheets (scale bar: 100 nm). **d** Lateral size distribution of Ti_3_C_2_ nanosheets. **e** TEM image of the CNTs (scale bar: 100 nm). **f** TEM image of the CNFs (scale bar: 200 nm). Inset: the photograph of CNFs dispersion. **g** Rheological behaviors of the fresh CNTs/CNFs ink and storage after 1 week. **h** Snapshots of the computational system at 0, 5, and 20 ns, respectively. **i** Variation of estimated density of the distance between the glucose molecule and the CNT surface. **j** Photographs of the CNTs, CCM-0.2, CCM-0.5, and CCM-1 film deposited on a cellulose membrane. **k** (i) Top‑view and (ii) cross‑sectional SEM images of CCM film (scale bar: 2 μm)

A typical chemical exfoliation process was used to prepare the Ti3C2 MXene nanosheets [42, 43], which was illustrated in Fig. S1. Briefly, ternary carbide MAX phase precursor (Ti_3_AlC_2_) was first employed to produce multilayered MXene after the selective removal of Al layer by using an aqueous LiF/HCl mixing solution. The obtained multilayered MXene showed a typical accordion-like structure (Fig. S2). After powerful sonication and subsequent centrifugation, the multilayered MXene could be separated into laminar Ti_3_C_2_ nanosheets. The successful removal of Al layer in the MAX phase was proved by the near disappearance of the peak at approximately 40.0° and the distinct shift of the (002) peak from 9.3° to 5.6° in the X-ray diffraction (XRD) spectrum (Fig. 2a). The transmission electron microscopy (TEM) image indicated that the Ti_3_C_2_ nanosheets possess a representative 2D lamellar structure (Fig. 2b). The finally obtained Ti_3_C_2_ nanosheets dispersion exhibited outstanding dispersity and hydrophilicity, reflected by the distinct Tyndall scattering effect. The successful fabrication of Ti_3_C_2_ nanosheets also could be proved by the X-ray photoelectron spectroscopy (XPS) spectra (Fig. S3). XPS results displayed the disappearance of Al element peak and the appearance of Ti-C (2*p*3) and Ti–O (2*p*3) peaks, which were consistent with our previous reports [44, 45]. In the atomic force microscopy (AFM) image, the individual Ti_3_C_2_ nanosheets showed an ultrathin thickness of around 1.5 nm (Fig. 2c). The mean lateral size distribution of the as-prepared Ti_3_C_2_ nanosheets was ~ 293.4 nm (Fig. 2d).

CNTs featured with a typical one-dimensional (1D) character were employed as a bridge to connect 2D Ti_3_C_2_ nanosheets. TEM image showed that the CNTs used in this article were about 33.19 nm in diameter and several micrometers in length (Fig. 2e). However, CNTs usually could not be assembled into macroscale structures with great uniformity due to the poor dispersibility of CNTs in an aqueous solution. CNFs, which could be extracted from softwood pulp via a 2, 2, 6, 6-tetramethylpiperidine-1-oxyl radical (TEMPO)-assisted oxidation process (Fig. S4a), were a promising 1D polymer that could be employed as an excellent surfactant due to their great amphiphilicity. As displayed in Fig. 2f, the CNFs solution with an obvious Tyndall scattering effect displayed well dispersibility. The Fourier transform infrared (FTIR) spectra showed distinct absorption peaks of O–H, COO^−^, and C = O of the as-prepared CNFs, testifying the successful oxidation process (Fig. S4b). The atomic force microscopy (AFM) images in Fig. S4c-d illustrated that CNFs possessed an average diameter of about 2.5 nm and a length of ~ 500 nm (200–600 nm range). CNTs powder was added into the CNFs dispersion and further ultrasonicated to prepare the CNTs/CNFs mixture. To investigate the stabilizing function of CNFs, CNTs/CNFs mixture with a CNTs/CNFs weight ratio of 10:1 was fabricated and found that the mixture displayed excellent stability over a week without any obvious precipitation (Fig. S5). The outstanding stability of CNTs/CNFs in water was mainly due to the associations between CNFs and CNTs, as reported by Hajian et al. [46]. The associations comprised mechanical wrapping, hydrophobic-hydrophobic interaction, and the fluctuation of the counter ions on CNFs [47]. Besides, the steric hindrance and the surface charges of CNFs were also the key factors for the great stability of CNTs/CNFs mixture. Figure 2g illustrates the corresponding rheological behavior of fresh and storage for 1 week of the CNTs/CNFs ink. Contrast to the fresh ink, the apparent viscosity of CNTs/CNFs ink after storage for 1 week showed almost no obvious change.

Molecular dynamic (MD) simulation was also conducted to further understand the formation mechanism of the CNTs/CNFs hybrid. Two glucose molecules were employed as a representative fragment of CNF to simplify the simulation process. As illustrated in Fig. 2h, CNFs were initially scattered around the CNT (0 ns) and were then gradually attached to the surface of the CNT at about 5 ns. Almost all the CNFs were stuck on the surface of the CNT till the end of simulation. To quantitively and intuitively investigate this process, the distance between the molecular chain of CNF and the CNT surface was further calculated. With the extension of simulation time, it could be seen that the high estimated density area (red area) of the glucose unit gradually concentrates to about 0.4 nm at about 5 ns and then remained relatively stable (Fig. 2i). At the same time, the results of histograms and estimated densities of the distance between CNF and CNT also verified this conclusion (Fig. S6). Moreover, as time elapsed, it could be seen that not only the distance gradually stabilized at about 0.4 nm, but also the angle simultaneously became ~ 0° (Fig. S7). These results indicated that, compared with the expensive and toxic surfactants, CNFs were a safer candidate for promoting dispersity of CNTs in aqueous solution, which were consistent with the previous reports [41, 48].

Compared with the poor stability of CNTs dispersion, CNFs/CNTs mixture displayed a favorable dispersibility (Fig. S8). Dispersion with different ratios of Ti_3_C_2_ nanosheets to CNFs/CNTs mixture was used to generate film structures by a vacuum-assisted filtration method employing a millipore filter membrane followed by drying in a thermocompressor. Hence, different hybrid films denoted as CCM-0.2, CCM-0.5, and CCM-1 had been designed and fabricated, respectively. The numbers represented the additive amount of the Ti_3_C_2_ nanosheets. For instance, “1” served as the additive amount was 1 mg. The CNTs filtered on membrane exhibited a fragile feature and poor uniformity owing to their poor dispersibility in water, whereas the obtained CCM film possessed an excellent uniformity and integrality (Fig. 2j). The energy-dispersive X-ray spectroscopy (EDS) of the CCM film indicating the uniform distribution of C, O, and Ti elements, which are the components of CNTs, CNFs, and Ti_3_C_2_ nanosheets (Fig. S9). Typical SEM images showed that the pristine MXene film displayed a regular nacre-like lamellar structure (Fig. S10). Figure 2k illustrates that 1D CNTs knitted the loose Ti_3_C_2_ nanosheets into a bridge interconnecting structure. Thus, the well-integrating structure of CNTs, CNFs, and Ti_3_C_2_ nanosheets would offer a continuous electronic pathway, endowing the CCM film with high elasticity and conductivity.

### Electromechanical Properties of CCM E-Skin

**Fig. 3:**
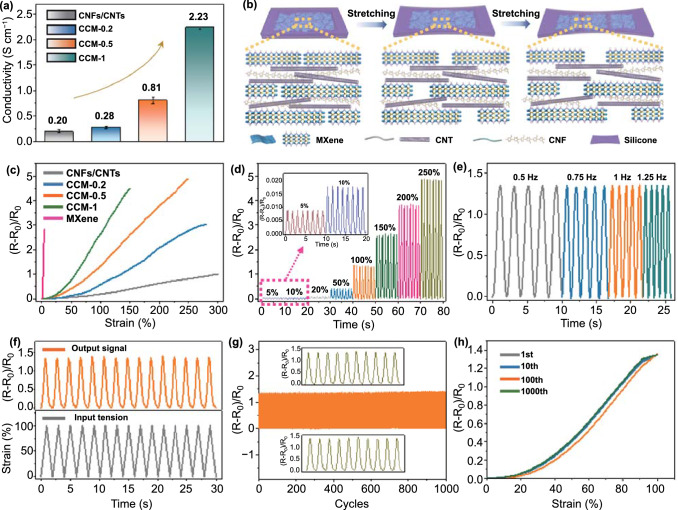
Electromechanical properties of CCM e-skin and mechanisms. **a** The electrical conductivity of the CCM e-skin at various MXene loadings. **b** Schematic diagram of the mechanism of the electromechanical responses of CCM e-skin. **c** Relative resistance changes of the CNFs/CNTs, CCM-0.2, CCM-0.5, CCM-1, and pristine MXene e-skin at different strains. **d** Relative resistance changes under various maximum stretching strains (5, 10, 20, 50, 100, 150, 200, and 250%) for the CCM-0.5 e-skin. **e** Relative resistance variation of the CCM-0.5 e-skin at different frequencies under 100% strain. **f** Time retention curves of the variation in resistance and strain with time. **g** Relative resistance change of the CCM-0.5 e-skin during 1000 cycles of stretching/relaxing between 0 and 100% strain at a constant frequency of 1 Hz. **h** Resistance variation for multiple-cycle tests: 1st (gray), 10th (blue), 100th (orange), and 1000th (green) cycles under 100% strain

We explored the conductivity of the CCM films with various Ti_3_C_2_ MXene nanosheets content by a four-point probe method. As depicted in Fig. 3a, the electrical conductivities of CCM films exhibited a typical MXene content-dependent behavior. With the increasement of MXene content, the conductivity of CCM film showed an obvious ascending trend and would achieve the highest value of 2.23 S cm^−1^ at a MXene additive amount of 1 mg. After transferring the CCM film onto the silicone rubber substrate, a flexible CCM e-skin could be achieved. The successful integration of 2D MXene nanosheets and 1D CNFs/CNTs endowed the flexible CCM e-skin with great potential for electromechanical responsing. Prior to stretching, the MXene nanosheets with typical 2D nanostructure and the 1D CNTs were interconnected with each other to generate a continuous electron conduction pathway (Fig. 3b). When the stretching starts, MXene nanosheets tended to slide with each other due to their weak van der Waals interactions [49, 50], whereas the CNTs could serve as “bridges” to connect MXene nanosheets. As the stretching continue, CNTs were pulled out and thus lead to the change of resistance of the CCM e-skin. The electromechanical responses of the CCM e-skin to tensile deformation had been studied by measuring the relative resistance variation ((*R* − *R*_0_)/*R*_0_) as a function of various strains. Figure 3c illustrates the relative resistance variation of various e-skins under an oriented tensile strain. It could be seen that the CNFs/CNTs e-skin exhibited a maximum working range of ~ 300%, but showed a low sensitivity and limited gauge factor (GF = (*R* − *R*_0_)/*R*_0_ε) (Fig. S11). Conversely, the pristine MXene e-skin demonstrated a high strain sensitivity, whereas its working strain was extremely low (~ 5%). Interestingly, the integration of 1D CNFs/CNTs and 2D MXene nanosheets endowed the CCM e-skin with wide sensing working range and high sensitivity, simultaneously. For example, the CCM-0.5 e-skin with a MXene additive amount of 0.5 mg possessed a broad working strain range of about 250% and a great sensitivity. Moreover, a clear trend showed that increase in MXene content could result in enhancement in sensitivity but reduction in stretchability for CCM e-skins.

Given that most human motion was irregular in movement amplitude and frequency, the systematic sensing evaluation of CCM e-skin had been implemented. The relative resistance variation of the CCM-0.5 e-skin under various cyclic strains was measured and is illustrated in Fig. 3d. At the maximum strains of 5, 10, 20, 50, 100, 150, 200, and 250%, the peak variations in the relative resistance were calculated to be 0.0087, 0.0177, 0.0774, 0.4367, 1.3481, 2.6457, 3.8524, and 4.8873, respectively, which was almost consistent with the corresponding results in Fig. 3c. It could be noted that the relative resistance response of the CCM-0.5 e-skin showed great repeatability under 10 stretching/relaxing cycles. The resistance signal increased as extend the applied strain and could almost fully recover as the strain relax in each cycle. Moreover, the CCM e-skin also exhibited a low hysteresis behavior under 100% strain (Fig. S12). Figure 3e shows that the relative resistance variation of the CCM-0.5 e-skin at different frequencies under 100% strain and almost no frequency dependence for the electrical response was founded. The electrical responses of the CCM-0.5 e-skin were very steady and remained stable as the stretching frequency increase from 0.5 to 1.25 Hz. Furthermore, the CCM-0.5 e-skin also possessed a fast response time (~ 150 ms, Fig. S13). The fast response would dramatically promote the real-time monitoring of fast and complicated movements. In addition, we also compared the output electric signals with the dynamic strain inputs. As illustrated in Fig. 3f, the output signals achieved a good match with the input strain waveform, demonstrating the outstanding response of the CCM e-skin to mechanical forces. Figure 3g displays the relative resistance variation of the CCM e-skin during 1000 stretching-releasing cycles between 0 and 100% strain at a constant frequency of 1 Hz. For 1000 cycles of stretching, the relative resistance changes of the CCM e-skin remained fairly stable. Moreover, the multiple sensing curves at the 1st, 10th, 100th, and 1000th showed a great coincidence with each other (Fig. 3h), indicating the excellent durability and long-term stability of the CCM e-skin.

**Fig. 4:**
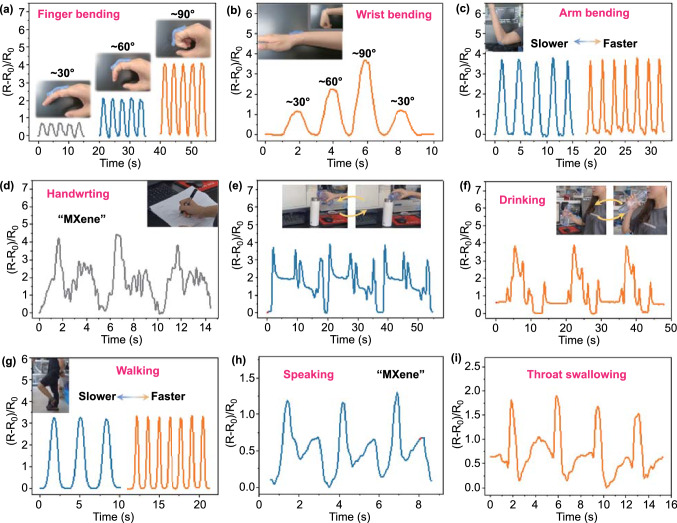
Response signal of CCM e-skin for the detection of various physiological movements. The monitoring process of **a** finger bending and **b** wrist bending for different angles. Inset: Photograph of a CCM e-skin attached to the index finger and the back of wrist. **c** Relative resistance response of CCM e-skin in detecting arm bending with different speeds. Inset: Photograph of a CCM e-skin attached to the arm joint. **d** Relative resistance response of CCM e-skin on handwriting “MXene.” Inset: Photograph of handwriting with CCM e-skin attached to the back of wrist. Detection of various arm movements, such as **e** pouring water into a cup and **f** drinking. **g** Relative resistance response of CCM e-skin in detecting waling with different speeds. Inset: Photograph of a CCM e-skin attached to the human knee. Responsive curves were recorded during **h** speaking “MXene” and i throat swallowing

The outstanding comprehensive performance, including superior flexibility, high sensitivity, great stability, and a wide stretching range, enabled the CCM e-skin to realize the real-time monitoring of full-range human activities, which involved large-scale motions and subtle physiological signals. We directly attached the CCM e-skin on various human joints and then sealed it with adhesive tape to detect large human body movements. For example, the CCM e-skins were mounted on a finger and wrist joints, respectively, to record the response signals during bending and relaxing motions (Fig. 4a-b). Diverse bending degrees could be accurately and quickly recognized by analyzing the relative change of the resistance. Moreover, the CCM e-skin was also able to monitor the arm bending motion with various bending frequencies (Fig. 4c). In addition to the regular joint bending motions, more complicated human activities including handwriting, pouring water into a cup, and drinking were also readily detected by adhering the CCM e-skin to the wrist or arm joints (Movies S1-S2 and Fig. 4d-f). Furthermore, the exceptional stretchability enabled the CCM e-skin to steadily detect the knee-joint bending motion, which required large tensile deformation. By attaching the CCM e-skin onto the knee joint, the leg movements such as walking and running could be readily detected by observing the variation in the relative resistance of the CCM e-skin in a highly repeatable manner (Figs. 4g and S14).

For the capture of subtle physiological signals, we mounted the CCM e-skin onto the human throat to detect the minor deformation of the epidermis and muscle. As expected, the CCM e-skin was capable of precisely recognizing various polysyllabic words, such as “MXene” and “sensor,” whose signal curves displayed two and one obvious peaks, respectively (Figs. 4h and S15). The remarkable phonation recognition ability endowed the CCM e-skin with significant possibility for the promising application in phonation rehabilitation exercises and intelligent artificial throat. Besides, the throat swallowing motion of the volunteer could also be detected in real-time (Fig. 4i). The CCM e-skin with impressive sensing capability for the accurate recognition of various human physiological signals and body motions via the electromechanical manner could realize the real-time supervision of full-range human activities.

### Joule Heating Performance of CCM E-Skin

**Fig. 5:**
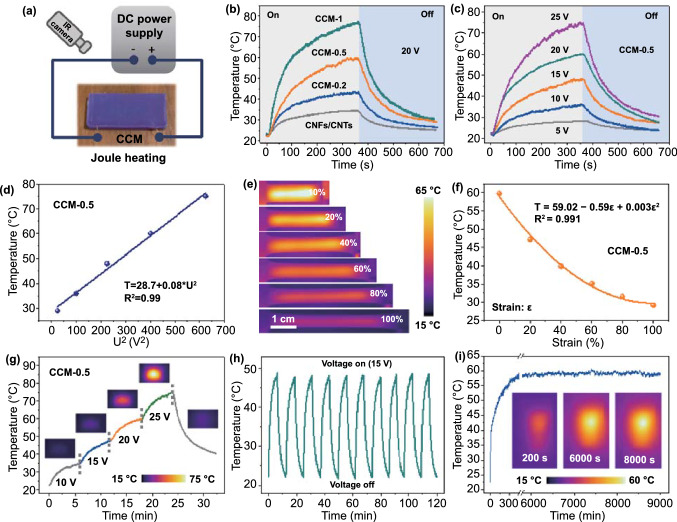
Joule heating performance of CCM e-skin by applying DC voltage. **a** Diagram of measurement setup for Joule heating using an IR camera. **b** Temperature profiles of CCM e-skin with various MXene content at an input voltage of 20 V. **c** Temperature profiles of CCM e-skin as a function of time at various input voltages. **d** The steady-state temperature of the CCM-0.5 e-skin as a function of the square of voltage. **e** Recorded temperature mapping of the CCM-0.5 e-skin at different strain levels. **f** Fitting curve of the variation in temperature with an initial temperature of 60 °C. **g** Temperature profiles of the CCM-0.5 e-skin under a stepwise increased voltage from 10 to 25 V. The insets display the thermal images of the CCM-0.5 e-skin at different voltages. **h** Heating stability test of the CCM-0.5 e-skin upon repeated applied voltage of 15 V. **i** Long-term temperature variation curve at an input voltage of 20 V for the electrical heaters CCM-0.5 e-skin. The insets are thermal images of the CCM-0.5 e-skin at 200, 6000, and 8000 s

For the CCM e-skin, the applied strain would generate a simultaneous variation in resistance, which offered the opportunity to realize dynamic Joule heating behaviors. To quantitatively understand the electrothermal properties of the CCM e-skin, we applied a direct current (DC) power system along the CCM e-skin and wirelessly monitored the temperature variation via a real-time infrared (IR) thermal imaging camera (Fig. 5a). Figure 5b illustrates the temperature–time curves of the CCM e-skin with various MXene content under an applied external voltage of 20 V. The CNFs/CNTs e-skin showed an inferior electrothermal performance with a low saturated temperature of about 34.5 °C. As increase the addition of MXene nanosheets, the Joule heating performance of CCM e-skins displayed an obvious enhanced tendency [51, 52]. For example, the CCM-1 e-skin with a MXene addition of 1 mg possessed a superior electrothermal conversion ability with an equilibrium temperature of approximately 77.4 °C. According to the Joule heating equation (*P* = *I*^*2*^*R* = *U*^*2*^/*R*), thermal power consumption was affected by the applied voltage and the resistance of the samples. As an example, the temperature profiles of the CCM-0.5 e-skin at various applied voltages are shown in Fig. 5c. A broad temperature range (28.4–74.7 °C) could be acquired at safely applied voltages below 25 V. As the applied voltage elevate from 10 to 15 and 25 V, the equilibrium temperature of the CCM-0.5 e-skin could increase from 36.2 to 48.4 and 74.7 °C, revealing a distinct upward tendency. Besides, the saturated temperatures of CCM e-skin in a steady state followed an obvious linear relation with the square of the applied voltage (Fig. 5d). This result revealed the leading role of the applied voltage in controlling the equilibrium temperature of CCM e-skin and was following previous reports [51, 53, 54].

Furthermore, the Joule heating performances of the CCM e-skin under tensile strain gradients were also explored by recording the time-dependent temperature. Figure 5e displays several IR thermographic photographs at various levels of mechanical strain under an input voltage of 20 V. Fitting was conducted to quantitatively illustrated the relationship between temperature and applied strain. The fitting formula was given as follows:

$${\text{T}} = 59.02 - 0.59\varepsilon + 0.003\varepsilon^{2}$$

as displayed in Fig. 5f, where T was the saturated temperature of CCM e-skin, *Ɛ* represented the applied strain. The equation suggested that the saturation temperature of the CCM e-skin exhibited an excellent nonlinear relationship with the applied strain, indicating the accuracy of the theoretical prediction of equilibrium temperature under various tensile strains. Figure 5g and Movie S3 shows the temperature profiles of the CCM e-skin under stepwise increased voltage from 10 to 25 V, and the insets depicted the corresponding thermal images. When the supplied voltage was gradually increased, the saturated temperature of CCM e-skin displayed a gradient upward trend, illustrating a similar result with the above-mentioned. Additionally, for the evaluation of the electrothermal stability of CCM e-skin, the recycling temperature variations of CCM e-skin were evaluated under 15 V applied voltage for about 6 min (on) and then decreased to room temperature (off) with natural cooling for 10 cycles. As shown in Fig. 5h, the Joule heating performance of the CCM e-skin did not exhibit any significant attenuation during the cycling process, confirming the excellent heating repeatability and recyclability. Moreover, the heating stability was also evaluated by recording the long-term time-dependent temperature upon the constant input voltage of 20 V. Figure 5i illustrates a very stable temperature of approximately 60 °C within the long duration of about 8500 s after attaining the equilibrium temperature, indicating an outstanding long-term heating stability of CCM e-skin.

### Thermomechanochromism Effects of CCM E-Skin

**Fig. 6:**
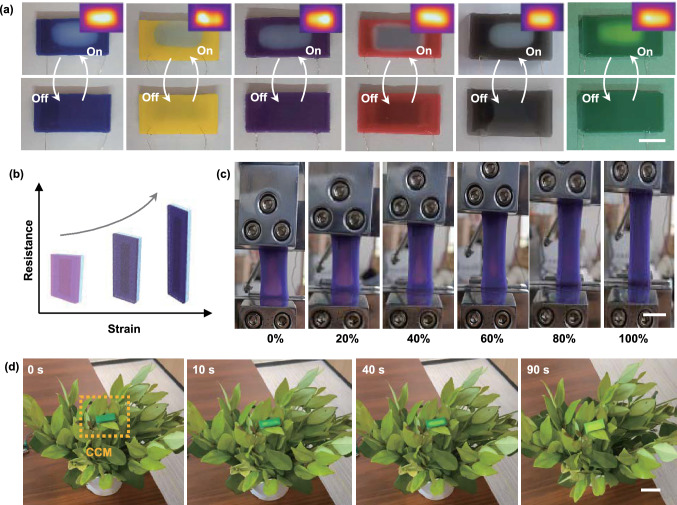
Thermomechanochromism effects and application example of the CCM e-skin. **a** CCM e-skins with various thermochromic species change color to white in response to an applied voltage of 20 V (scale bar: 1 cm). **b** Diagram shows the variation tendency of the resistance and color of the CCM e-skin under constant stretching. **c** Photographs of the CCM e-skin being stretched while maintaining constant applied voltage (scale bar: 1 cm). **d** Thermochromic application example of the CCM e-skin for military camouflage by switching on/off the voltage (scale bar: 2 cm)

To visualize the heat response of CCM e-skin to the tensile strain, we utilized a composite of thermochromic pigments dispersed in silicone rubber, which could reflect the variation of strain, resistance, and temperature of CCM e-skin based on the color change. The intermediate CCM film was used as a temperature-tunable heater with applied mechanical strain, whereas the silicone rubber mixed with thermochromic pigments could serve as an encapsulation and temperature display. As shown in Fig. 6a, various thermochromic pigments were mixed with liquid silicone rubber to generate a uniform discoloration layer. The CCM e-skin with thermochromic pigment maintained its initial color (such as blue, yellow, purple, and red) at room temperature but would change to white beyond 31 or 65 °C (black to white). Moreover, given that the thermochromic components possess various response temperatures, mixing two kinds of pigments may generate a new system that exhibited three color states and presented a wider and more plentiful pallet (Fig. S16a-b and Movie S5). Besides, the discoloration area of the CCM e-skin could also be controlled by the regulation of applied voltages (Fig. S16c). The capability to change the color of a specific area indicated this approach may be suitable for application in soft, passive displays.

In addition, when increasing the applied strain, the Joule heating behaviors of CCM e-skin would display an attenuation trend, as shown conceptually in Fig. 6b. Figure 6c illustrates photographs of the color change of CCM e-skin at various strains. In the initial state, the CCM e-skin could be rapidly heated and change the color from purple to pink on account of the successful activation of the purple thermochromic components. With the applied strain increasing gradually, the color of the CCM e-skin changed from pink to its original color (purple) due to the variation of saturation temperature. This demonstration displayed the potential feasibility of realizing visual strain sensing by detecting the multiple, step-wise color change during stretching. Especially, unlike the traditional strain-sensing color-changing materials, which were mainly based on the complicated molecular-designing strategies, the theory applied in this work was more general since it did not reply on mechanochemistry. Thus, the outstanding heating capability and thermochromic performance allowed the CCM e-skin to be a promising candidate for military camouflage. For instance, the CCM e-skin could be attached onto the equipment or skin of soldiers to escape from the enemies by changing color, just like the chameleon. As illustrated in Fig. 6d and Movie S6, the CCM e-skin changed color from dark green to light green within 90 s at a constant voltage of 20 V. Obviously, the light green would be more helpful for the CCM e-skin to disguise in green plants or forests.

### CCM E-Skin for Visual Motion Monitoring

**Fig. 7:**
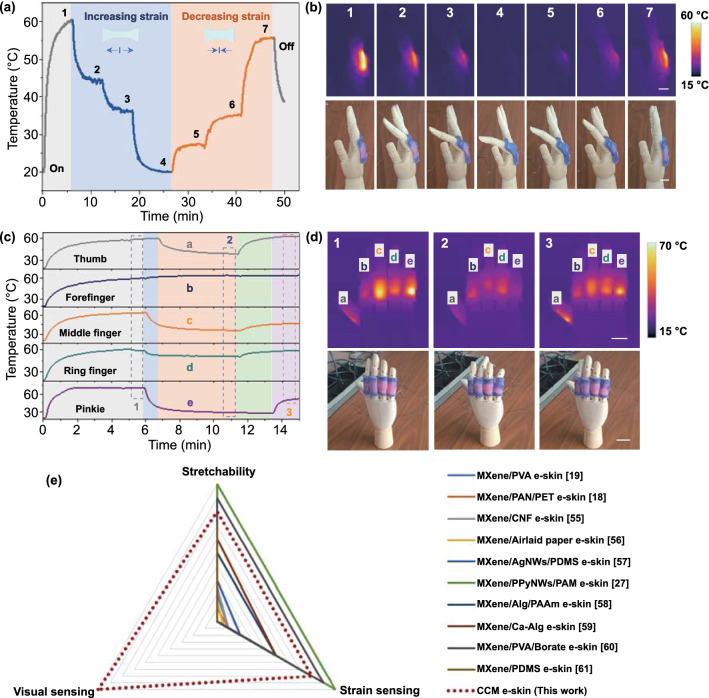
An interactive temperature-/color-changing e-skin for visual motion monitoring. **a** Temperature variation of the CCM e-skin with gradually bending of finger and corresponding recovery process. **b** Photographs of the manipulator and corresponding temperature distribution images, corresponding to various bending states of the finger in **a** (scale bar: 1.5 cm). **c** Temperature variation with the movement of each finger. **d** Photographs and corresponding temperature distribution images of fingers under different states of motion in **c** (scale bar: 2 cm). **e** An overall performance comparison of the CCM e-skin with other MXene-based flexible e-skins. The numbers in **e** are the serial number of the references

The CCM e-skin with excellent temperature/color sensibility to various tensile strains possessed a significant potential for the application in visual human activities monitoring. Here, the CCM e-skin was attached onto an index finger joint of the hand model and the real-time temperature, and color changes of them under different strains were recorded. As shown in Fig. 7a-b and Movie S7, the CCM e-skin, which was adhered to the finger, could change color from purple to pink and achieve an equilibrium temperature of about 60 °C under an input voltage of 20 V. Subsequently, it could be seen that the temperature of the CCM e-skin displayed a distinct gradient descending tendency and reached a steady-state eventually with the gradual increase in bending degree of a finger. Correspondingly, the CCM e-skin changed to its initial color of purple from pink. Especially, the color of the CCM e-skin could gradually change to pink again, when the finger recovered to its original relaxed state. And the temperature also exhibited an obvious gradient escalating trend corresponding to the color change. Thus, the finger bending motion could be visually detected by analyzing the change of temperature and color of the CCM e-skin. In addition, the CCM e-skin also displayed a similar variation trend of the temperature and color during monitoring wrist bending motion, indicating the general applicability of CCM e-skin (Fig. S17 and Movie S8). Thus, color variation is an important performance for the smart e-skins. Compared to the imperceptible human motions with small strain, color variation will be a more intuitive and perceptive signal for the accurate monitoring of full-range human activities.

Based on the single-channel sensing above, we also explored a visual multichannel sensing system comprising of five CCM e-skins working in a parallel connection and monitored the individual fingers bending of the manipulator with great accuracy. Figure 7c-d shows that all the five CCM e-skins could achieve a saturated temperature of approximately 60 °C and changed their colors from purple to pink at a safely applied voltage of 20 V. Subsequently, the temperature of CCM e-skins showed an obvious decreasing trend as the fingers bended to a greater angle. Meanwhile, the CCM e-skins changed to different colors correspondingly. Therefore, the highly stretchable and thermochromic e-skin based on the conductive CCM film could be used as a wearable strain sensor to realize the wireless and visual monitor of human motions. Additionally, an overall performance comparison of the CCM e-skin with other MXene-based e-skins was also presented [18, 19, 27, 55–60]. As shown in Fig. 7e, the CCM e-skin possessed an excellent comprehensive performance, such as the remarkable mechanical property and the outstanding sensing performance. Especially, compared with other MXene-based e-skins without the visual sensing capability, the CCM e-skin could synchronously achieve digital electrical response and optical visualization to external mechanical stimulus. To the best of our knowledge, there was lack of the MXene-based multimodal fusion strategy until we proposed in this work. The simple design philosophy and reliable operation of the demonstrated e-skin were expected to provide an ideal platform for next-generation flexible electronics.

## Conclusions

In summary, this paper demonstrated a bioinspired flexible e-skin that integrates electromechanical sensing and optical display to achieve visual motion monitoring. This platform was simple, only composed of a conductive CCM layer and a thermochromic silicone-based elastomer layer. The incorporation of 2D MXene and 1D CNTs endowed the CCM skin with remarkable strain-sensing ability via analyzing the resistance variation during deformation. Interestingly, benefiting from this striking electromechanical sensing feature, the CCM e-skin was capable of achieving the real-time monitoring of human activities, such as handwriting, drinking, walking, and speaking. Besides, the CCM film with exceptional Joule heating performance could deliver their thermal energy to the elastomer layer and consequently trigger the color variation of thermochromic pigments. By regulating the applied voltage and the combination of multiple pigment species, the CCM skin could realize a wider range and dynamic coloration for passive displays and military camouflage. More importantly, CCM e-skin attached to the joints under a constant input voltage could undergo a color change with various joint movement behaviors offering a visualization of motion recognition function. We believe that such simple fabrication and remarkable operation of this bioinspired e-skin will open a new chapter for the design of novel flexible electronics, which would broaden its application in various fields, such as wearable devices, human–machine interactions, and soft intelligent robots.

## Supplementary Information

Below is the link to the electronic supplementary material.

Supplementary file1 (PDF 854 KB)

Supplementary file2 (AVI 10812 KB)

Supplementary file3 (AVI 9292 KB)

Supplementary file4 (AVI 5748 KB)

Supplementary file5 (AVI 12005 KB)

Supplementary file6 (AVI 7842 KB)

Supplementary file7 (AVI 8497 KB)

Supplementary file8 (AVI 5728 KB)

Supplementary file9 (AVI 3534 KB)
