# Supplementary material for: Bioinspired MXene-Based User-Interactive Electronic Skin for Digital and Visual Dual-Channel Sensing
Source: Nanomicro Lett. 2022 May 3;14:119. doi: 10.1007/s40820-022-00838-0 (PMC9065218; doi:10.1007/s40820-022-00838-0)
Supplement: Supplementary file 1 — Supplementary file1 (PDF 854 KB) [file 40820_2022_838_MOESM1_ESM.pdf]

Supporting Information for

Bioinspired MXene-Based User-Interactive Electronic Skin for Digital and Visual Dual-Channel Sensing

Wentao Cao[1, 2], Zheng Wang[1], Xiaohao Liu[1], Zhi Zhou[1], Yue Zhang[1], Shisheng He[1, *], Daxiang Cui[1, 2, 3, *], Feng Chen[1, *]

[1]Department of Orthopedic, Spinal Pain Research Institute, Shanghai Tenth People's Hospital, School of Medicine, Tongji University, Shanghai 200072, P. R. China

[2]National Engineering Research Center for Nanotechnology, Shanghai 200241, P. R. China

[3]Institute of Micro-Nano Science and Technology, School of Electronic Information and Electrical Engineering, Shanghai Jiao Tong University, Shanghai 200240, P. R. China

*Corresponding authors. E-mail: fchen@tongji.edu.cn (Feng Chen); tjhss7418@tongji.edu.cn (Shisheng He); dxcui@sjtu.edu.cn (Daxiang Cui)

Supplementary Figures

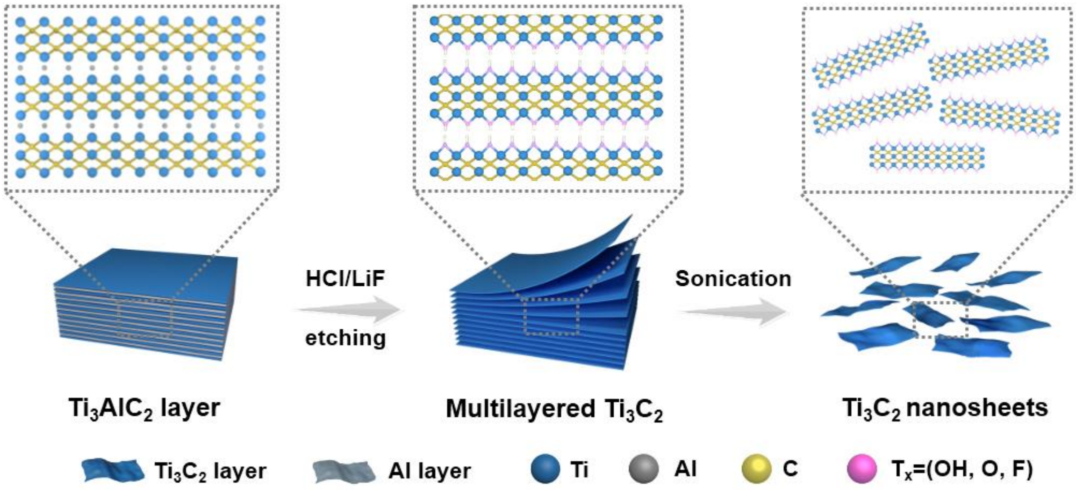

Fig. S1 Schematic for the synthesis of Ti_3C_2 MXene

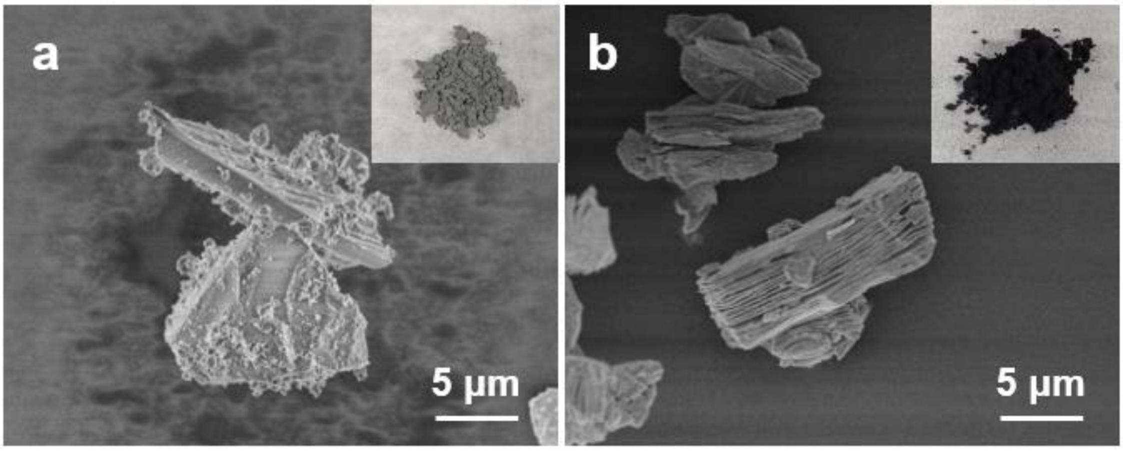

Fig. S2 SEM images of (**a**) Ti_3AlC_2 and (**b**) accordion-like multilayered MXene

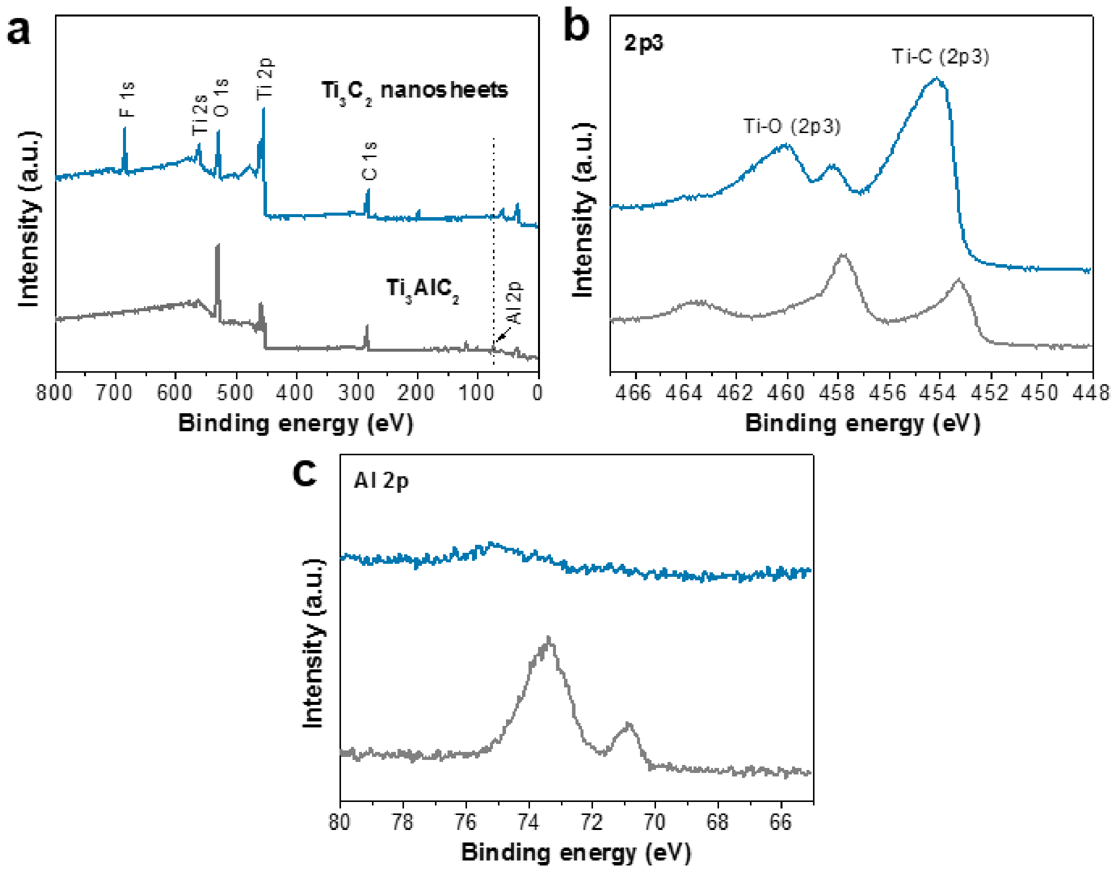

Fig. S3 (**a**) XPS survey, (**b**) Ti 2p, and (**c**) Al 2p spectra of Ti_3AlC_2 precursor and Ti_3C_2 MXene nanosheets

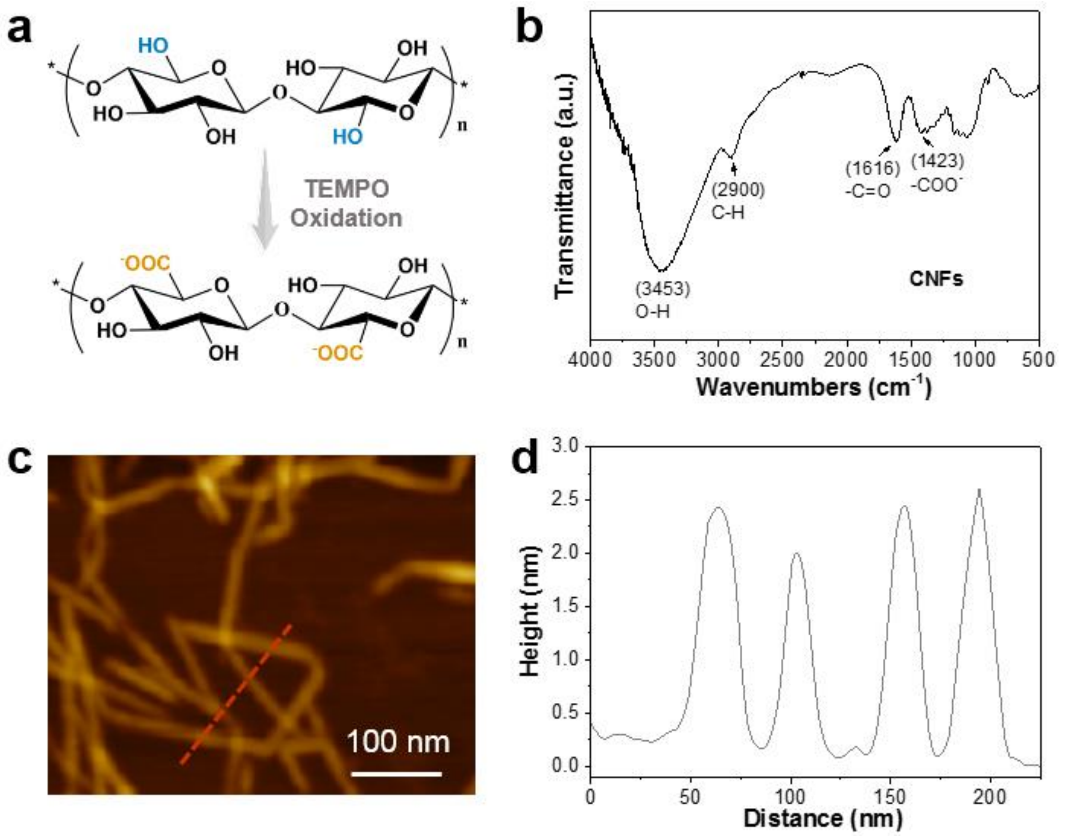

Fig. S4 (**a**) Schematic of the TEMPO oxidation process to prepare CNFs. (**b**) FTIR spectra, (**c**) AFM image, and (**d**) height profiles of CNFs

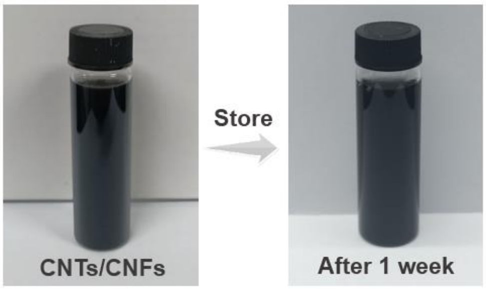

Fig. S5 CNTs/CNFs solution stability test over a week

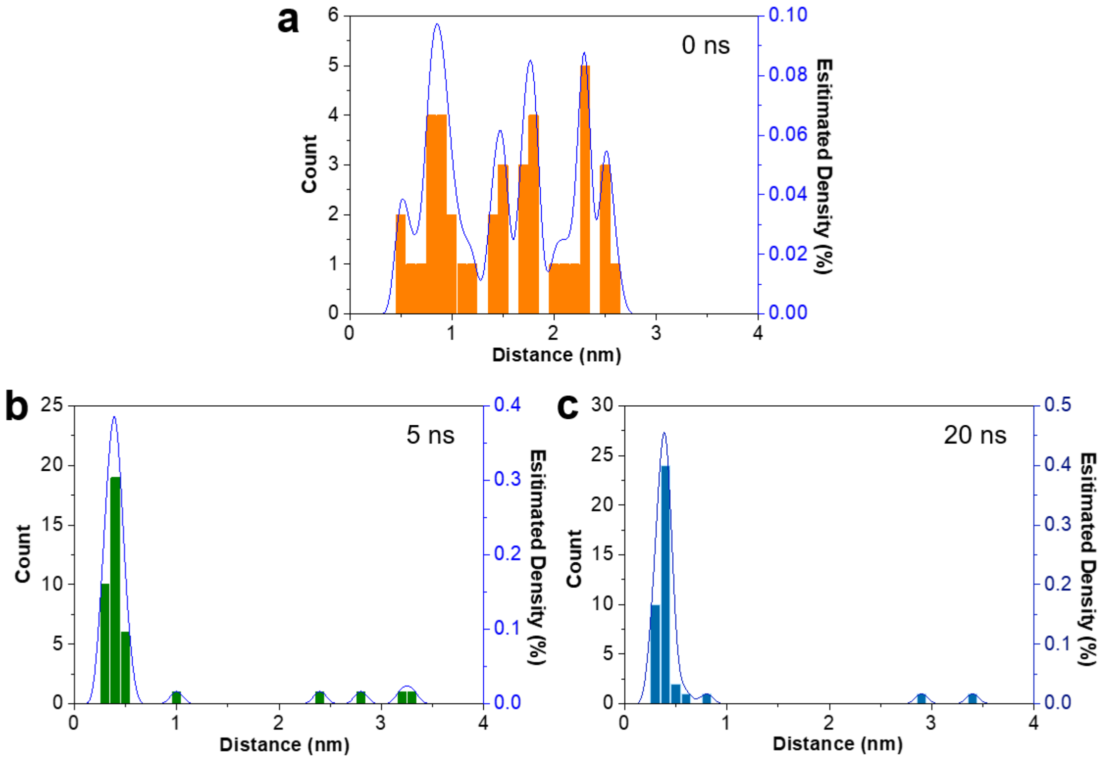

Fig. S6 Histograms and estimated densities of the distance between the atoms of glucose and the surface of CNT at (**a**) 0, (**b**) 5, and (**c**) 20 ns

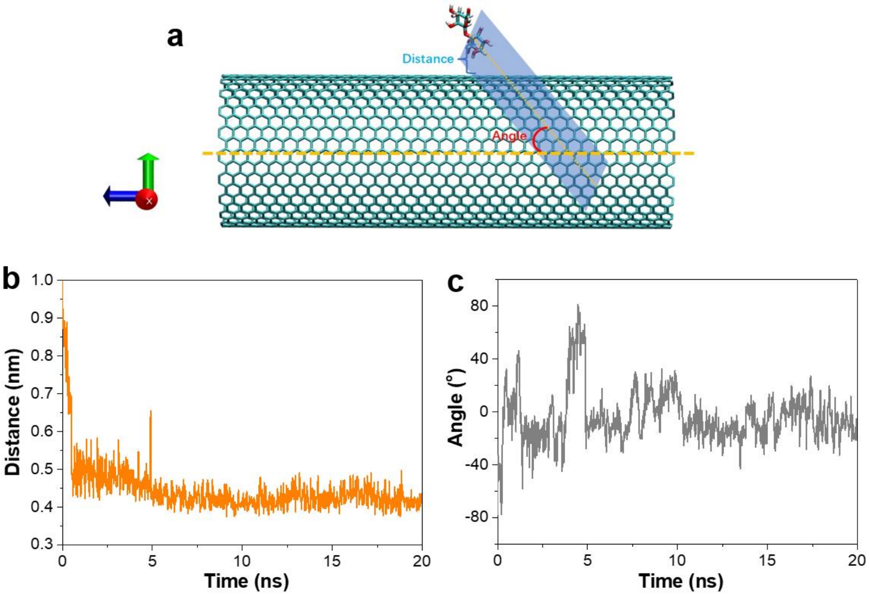

Fig. S7 (**a**) Definition of the parameters between glucose and CNT. Evolution of (**b**) the distance and (**c**) the angle between glucose and CNT

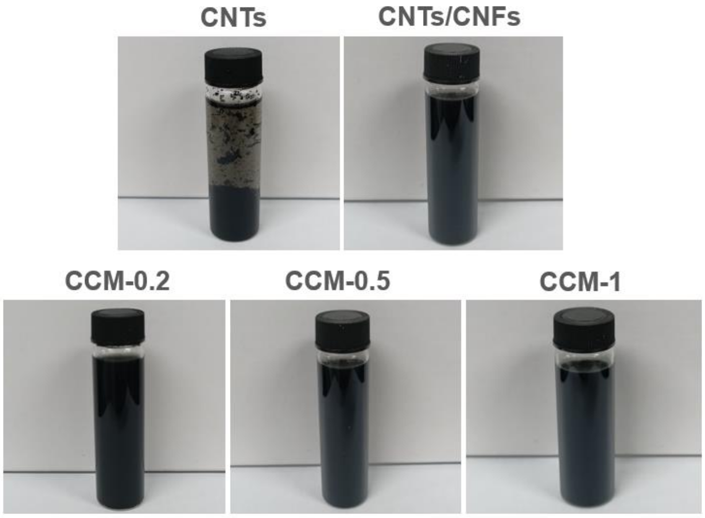

Fig. S8 Digital photographs of CNTs, CNTs/CNFs, and CCM dispersion

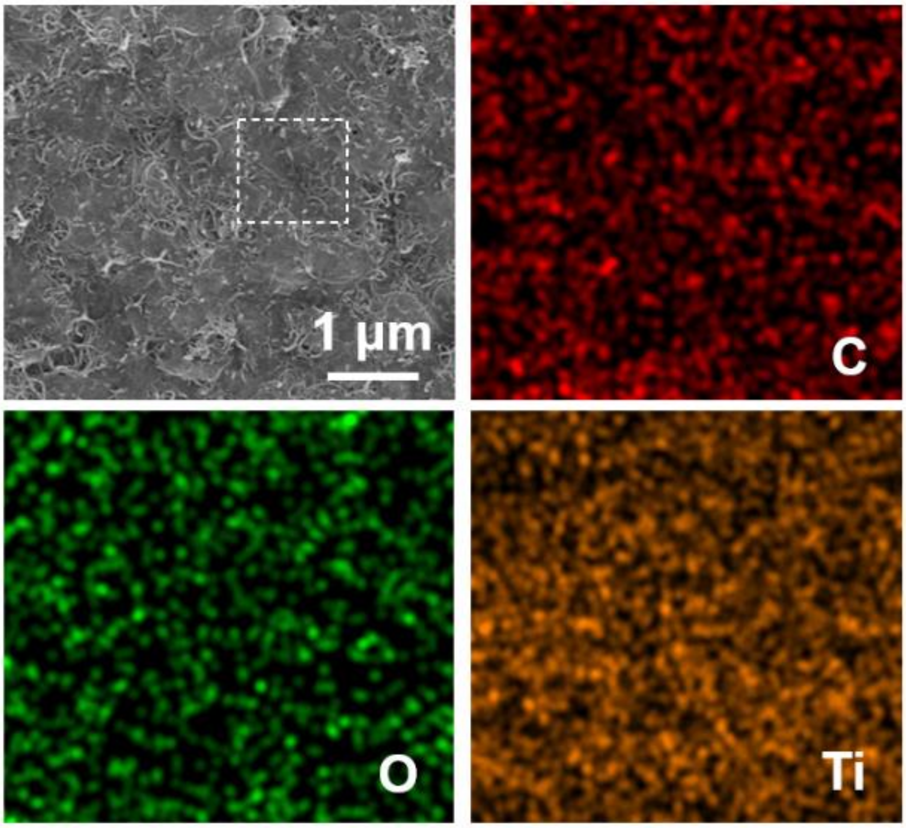

Fig. S9 EDS elemental mapping image of the CCM film

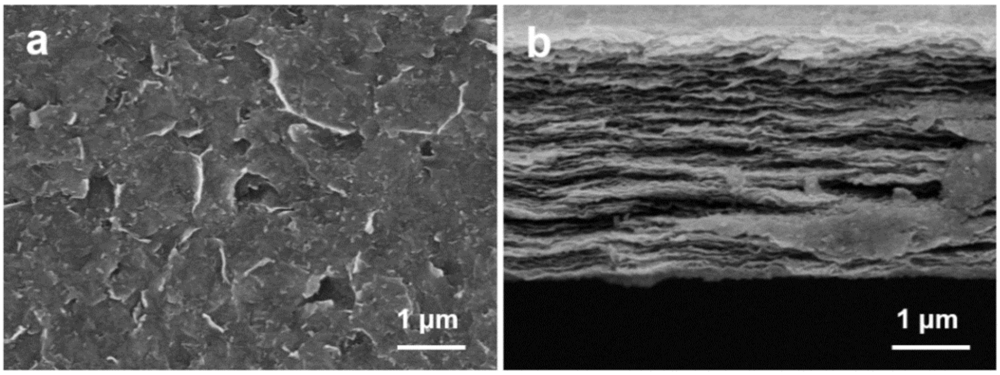

Fig. S10 (**a**) Top-view and (**b**) cross-sectional c

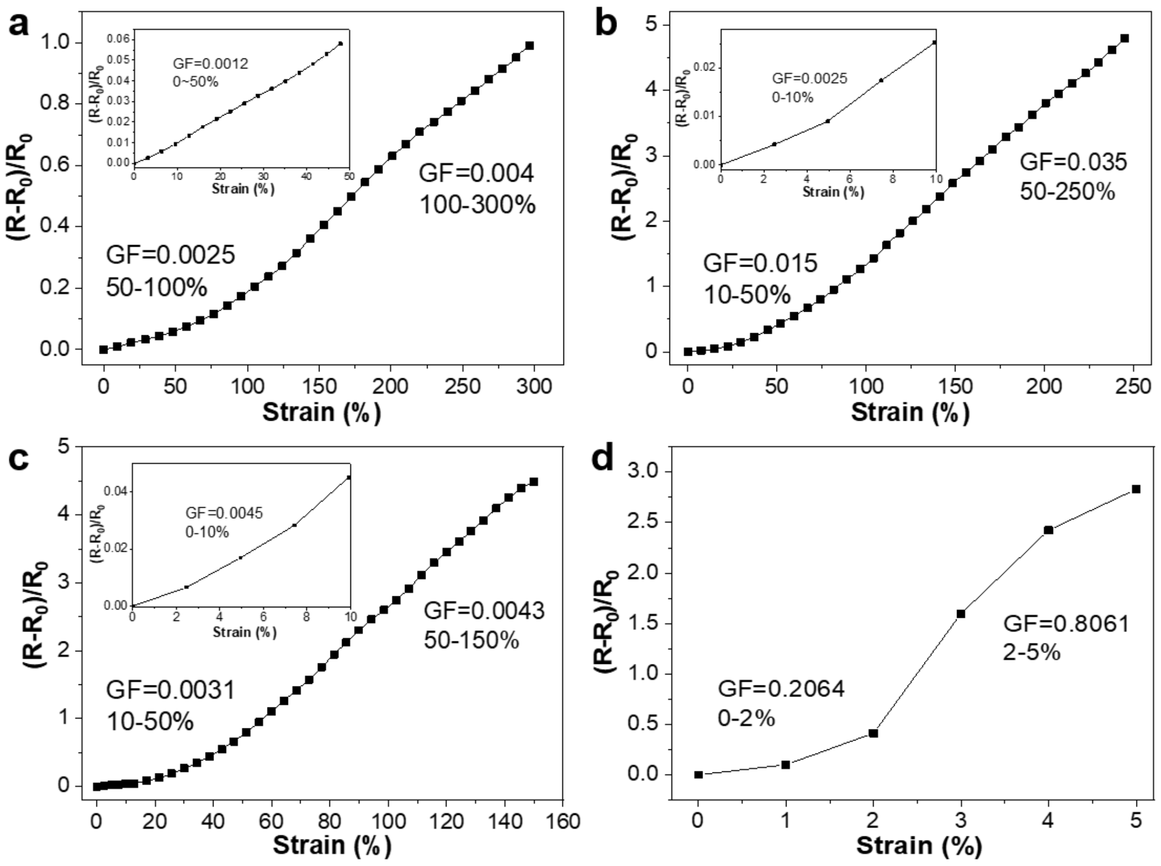

Fig. S11 The GF and linear behavior of (**a**) CNFs/CNTs, (**b**) CCM-0.5, (**c**) CCM-1, and (**d**) pristine MXene e-skin

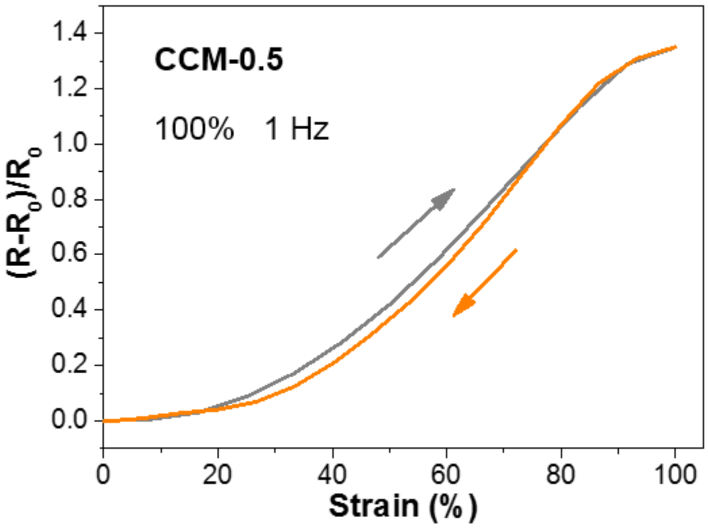

Fig. S12 Typical relative resistance changes versus applied strain curves under one stretch and release cycle for the CCM-0.5 e-skin at a frequency of 1 Hz under 100% strain

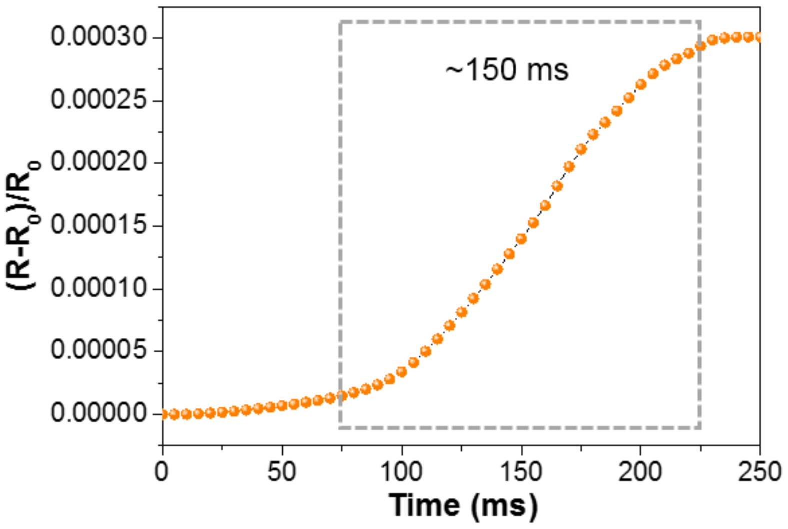

Fig. S13 Relative resistance changes of the CCM-0.5 e-skin under a quasi-transient step strain of 0.5%

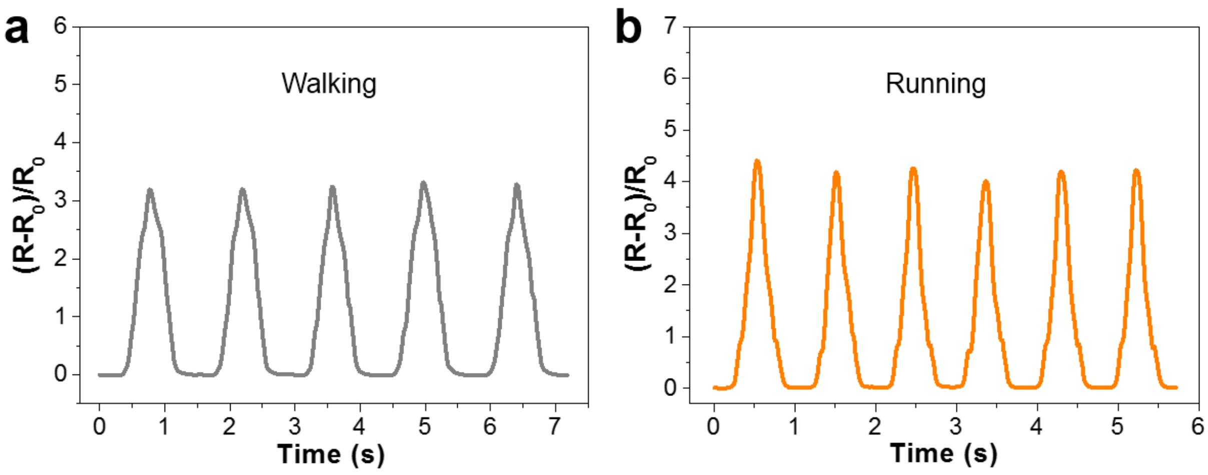

Fig. S14 Responsive curves of the CCM-0.5 e-skin on the knee under motions of (**a**) walking and (**b**) running

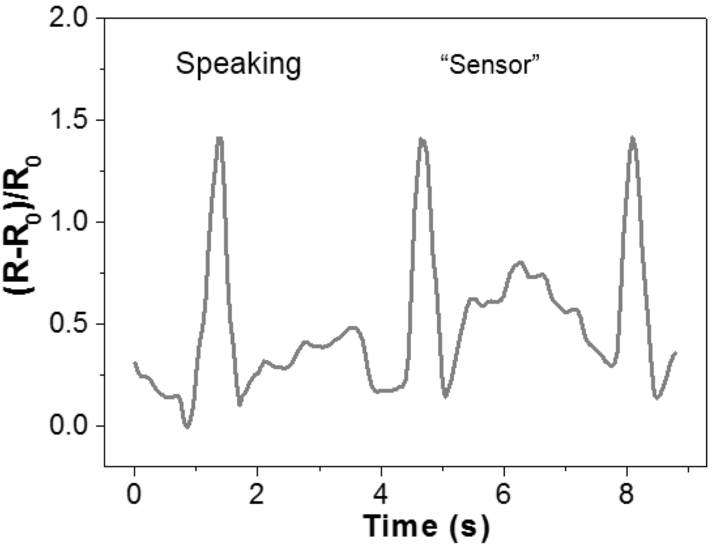

Fig. S15 Responsive curve recorded during speaking "sensor"

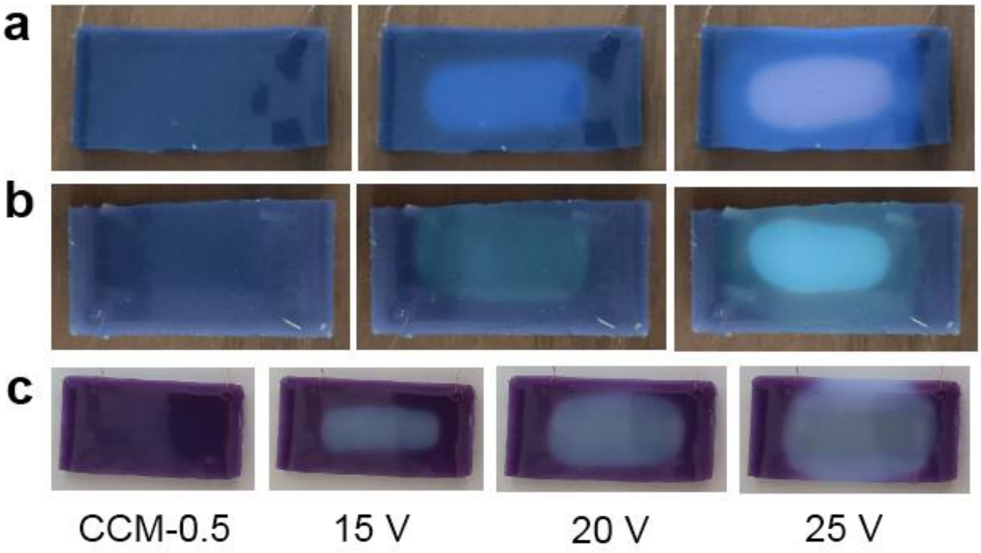

Fig. S16 (**a**) Photographs of a sample with black and blue pigments that change color in response to an applied voltage of 20 V. (**b**) Photographs of a sample with black and purple pigments that change color in response to an applied voltage of 20 V. (**c**) Photographs of a sample with purple pigments that change color in response to various applied voltage

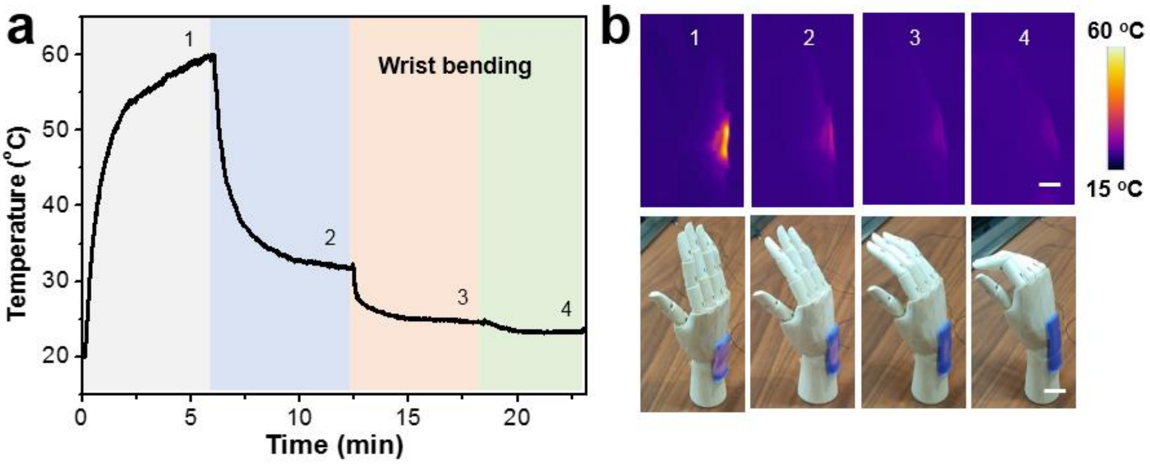

Fig. S17 (**a**) Temperature variations of the CCM e-skin with gradually bending of wrist. (**b**) Photographs and corresponding temperature mapping of wrist under different states of motion in a) (scale bar: 2 cm)
